# *Caenorhabditis elegans* expressing a Vitellogenin::GFP fusion protein show reduced embryo content and brood size

**DOI:** 10.17912/micropub.biology.000532

**Published:** 2022-03-01

**Authors:** Emily A. Erdmann, Olivia Abraham, Heather A. Hundley

**Affiliations:** 1 Department of Biology, Indiana University, Bloomington, IN, USA; 2 Medical Sciences Program, Indiana University School of Medicine- Bloomington, Bloomington, IN, USA

## Abstract

Vitellogenin::GFP fusion proteins have been used in several studies of the synthesis, endocytosis, and function of yolk in *Caenorhabditis elegans*. Here we report that one commonly used transgenic strain harboring a *vit-2::gfp* fusion displays defects in reproduction that lead to a significantly decreased embryo content and brood size in adult worms.

**Figure 1. The effect of the vit-2::gfp transgene on embryo content and brood size:**
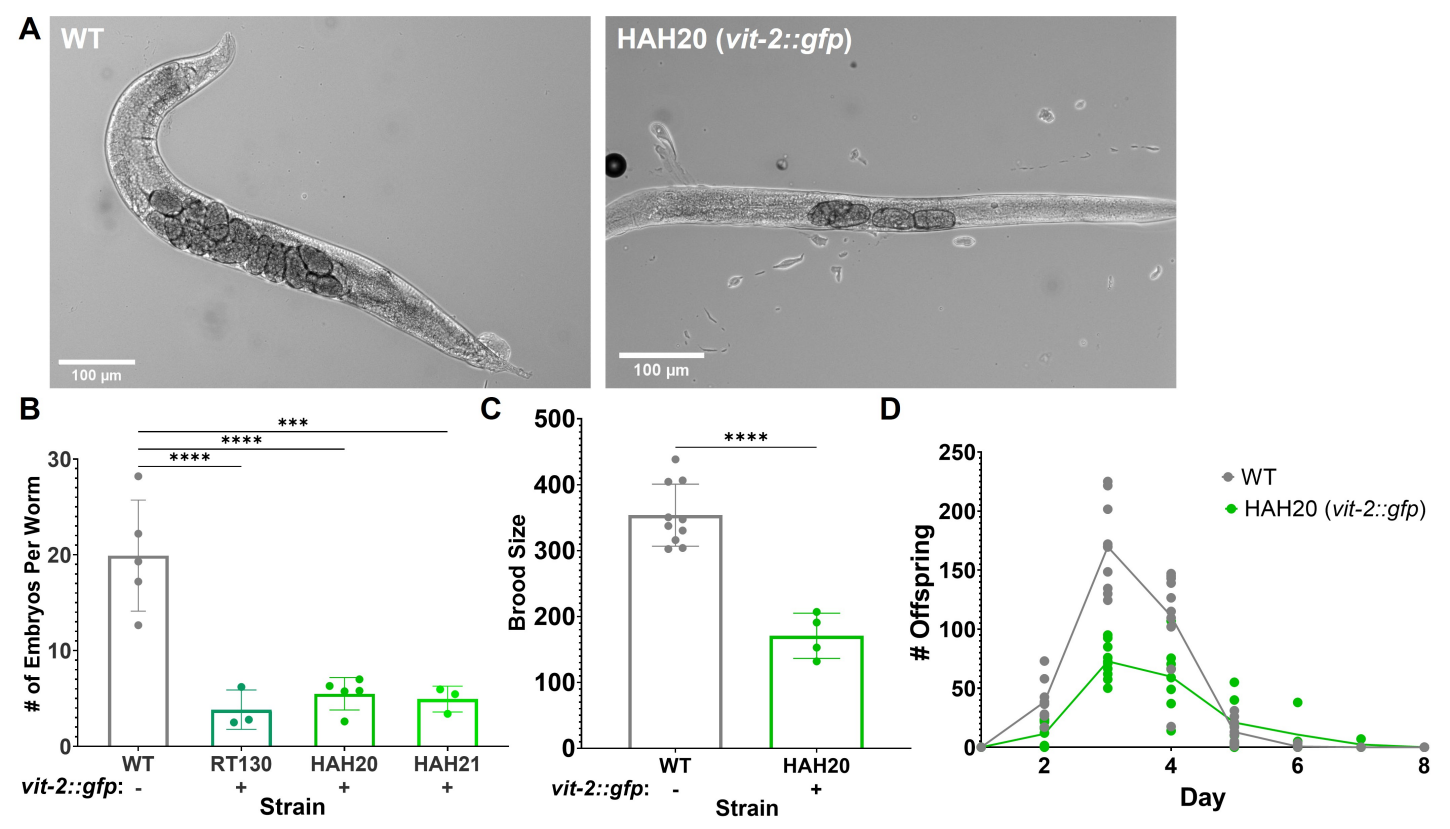
A) Representative DIC images of developmentally synchronized wild-type (WT) and VIT-2::GFP worms. Scale bar = 100 μm. B) Synchronized adult worms from the indicated strains were individually placed in bleach to dissolve the cuticle, and the embryos contained in each worm were counted. Each datapoint represents the average number of embryos per worm from an individual biological replicate of twenty worms. Error bars represent standard error of the mean (SEM). Statistical significance determined by one-way ANOVA. C, D) Synchronized adult worms of the indicated genotypes were moved to fresh NGM plates seeded with *Escherichia coli* OP50 each day. The offspring on each plate were counted for two consecutive days following hatching. The number of offspring in total (C) and per day (D) were plotted. Datapoints represent technical replicates (with two worms each) for the two biological replicates performed. C) Error bars represent SEM. Statistical significance determined by t-test. **** *p* value ≤ 0.0001, *** *p* value ≤ 0.001. D) Day 0 represents L4 stage worms.

## Description

Vitellogenins are the most conserved family of yolk proteins in oviparous animals, wherein the proteins are typically synthesized in extraovarian organs, secreted to fluid of a circulatory system and taken up by the ovary (Ramos *et al.* 2022). In *C. elegans*, vitellogenins are synthesized in the intestine, transported into the body cavity, and taken up by developing oocytes through receptor-mediated endocytosis to provide a source of nutrients for the developing embryo (Grant and Hirsh 1999; Hall *et al.* 1999; Kimble and Sharrock 1983). *C. elegans* has six vitellogenin genes (*vit-1* – *vit-6*), which combine to make up four major yolk proteins: YP170A and YP170B, YP115, and YP88 (Perez and Lehner 2019). To visualize the process of yolk uptake by oocytes, many published studies have made use of worms expressing an integrated transgene coding for a fusion protein of green fluorescent protein (GFP) with VIT-2, the most abundantly expressed *C. elegans* vitellogenin (Grant and Hirsh 1999). This fusion protein construct has been used in several studies as not only a means of visualizing yolk uptake via receptor-mediated endocytosis, but also as a means of quantifying embryo content and brood size in a variety of genetic backgrounds (Balklava *et al.* 2016; Grant and Hirsh 1999; Van Rompay *et al.* 2015). Here we report that wild-type strains expressing this VIT-2::GFP construct have a phenotype of reduced embryo production, resulting in fewer embryos contained in adult animals at any time as well as a markedly reduced brood size compared to N2 animals. We urge future studies to consider the effects of the *vit-2::gfp* transgene on normal reproduction when making conclusions about data generated using these strains, and to compare all experimental findings to a wild-type strain where possible.

Upon using *pwIs23* [*vit-2::gfp*] expressing worms (RT130) in our own experiments, a discrepancy in the size of young adult VIT-2::GFP worms compared to wild-type N2 worms was noted, as well as a difference in the number of worms on each plate during routine passaging. To more accurately assess whether these differences were due to variations in strain backgrounds, RT130 was back-crossed in our laboratory, and wild-type worms expressing the VIT-2::GFP transgene were obtained. Upon inspection of these individual worms, the VIT-2::GFP worms appeared to contain fewer embryos *in utero* than wild-type worms **(Figure 1A)**. To quantify the apparent differences in embryo content, an egg counting assay was performed where synchronized day one adult worms were dissolved individually in 20% bleach solution. Upon dissolution of the cuticle, the remaining embryos from each worm were counted. For RT130 (Grant and Hirsh 1999) as well as our two independently crossed VIT-2::GFP strains (HAH20 and HAH21), the embryo count per worm was about half to one-third that of wild-type N2 worms **(Figure 1B)**.

To determine whether the observed differences in worm population during passaging were the result of a decreased brood size, the total brood size of wild-type and VIT-2::GFP (HAH20) worms over their reproductive adulthood was determined. Two synchronized L4 worms were plated on each of 4 plates per strain and moved to fresh plates each day. Offspring on each plate were counted for two consecutive days after removal of the adult worms. Mirroring the embryo content, VIT-2::GFP worms produced about half as many offspring during their lifetimes as wild-type worms **(Figure 1C)**. As egg-laying for both strains started, peaked, and finished on the same days **(Figure 1D)**, it is unlikely that the differences in embryo content/brood size result from either a difference in egg-laying behavior or a developmental delay, but rather reflect an overall reduction in embryo production throughout the lifetime of VIT-2::GFP worms.

While the mechanism behind this reduction in brood size is still unclear, it is possible that the unusual uptake of VIT-2::GFP by coelomocytes may be related (Paupard *et al.* 2001). Looking back at the literature, one study compared the reproductive output of *vit-2::gfp* worms to wild-type animals (Seah *et al.* 2016). The supplemental data in that study indicates the brood size of RT130 was approximately 50 worms less than a wild-type strain, but the assay did not reach statistical significance. Our analysis of several strain backgrounds and quantification of 50-100 worms in multiple assays revealed a significant brood size defect, which we feel is important to keep in mind for those using VIT-2::GFP strains in future studies. We also feel that conclusions from past studies that did not verify results from VIT-2::GFP strains in wild-type worms or include a non-GFP control should be cited with caution or verified accordingly.

## Methods

*Worm strains and maintenance:* All worm strains were maintained at 20⁰C on nematode growth media (NGM) seeded with *Escherichia coli* OP50. Worms were thawed regularly from frozen stocks to minimize effects of accumulated random mutations.

*DIC imaging:* Worms were synchronized by plating 20 gravid adult worms on NGM plates seeded with *E. coli* OP50 for 2 hours before removal of all adults. The synchronized embryos were allowed to develop to day 1 adulthood. Live worms were mounted on 2% agar in microinjection oil before imaging with a Zeiss AXIO Observer at 10x.

*Embryo counting assay:* Adapted from: (Hart 2006). Worms were synchronized as stated above and allowed to develop to day 1 adulthood. 20 worms from each strain were placed in individual drops of 20% bleach, and the cuticles were allowed to dissolve for ~10 minutes. The remaining embryos in each bleach drop were counted. The average embryo count from each set of 20 worms was calculated, representing one biological replicate. 3-5 biological replicates were performed for each strain.

*Brood size assay:* Adapted from: (Reich *et al.* 2018; Yen and Curran 2020). Worms were synchronized as stated above. Two L4-stage worms of each strain were plated on each of 10 NGM plates seeded with *E. coli* OP50. The worms were moved to fresh plates each day for the remainder of the experiment. Offspring laid on each plate were counted for two subsequent days after removal of the adults and divided by the number of adult worms plated on that plate, with adjustments made for worms that died or went missing during the experiment. The experiment was ended when all worms produced no offspring for two consecutive days.

## Reagents

**Table:** 

**Strain:**	**Genotype:**	**Available from:**
N2	wild-type	CGC
RT130	RT130; *pwIs23* [*vit-2::gfp*]	CGC
HAH20	N2; *pwIs23* [*vit-2::gfp*]	HAH lab
HAH21	N2; *pwIs23* [*vit-2::gfp*]	HAH lab
